# Characteristics of risk factors for acute kidney injury among inpatients administered sulfamethoxazole/trimethoprim: a retrospective observational study

**DOI:** 10.1186/s40780-022-00251-0

**Published:** 2022-08-01

**Authors:** Yuki Shimizu, Toshinori Hirai, Yukari Ogawa, Chihiro Yamada, Emiko Kobayashi

**Affiliations:** 1 Department of Pharmacy, Tokyo Women’s Medical University Yachiyo Medical Center, 477-96, Owada-shinden, Yachiyo, Chiba 276-0046 Japan; 2 Department of Pharmacy, Faculty of Medicine, Mie University Hospital, Mie University, 2-174, Edobashi, Tsu, Mie 514-8507 Japan; 3 Department of Pharmacy, Faculty of Pharmacy, Musashino University, 1-1-20 Shin-machi, Nishitokyo, Tokyo, 202-8585 Japan

**Keywords:** Sulfamethoxazole, Trimethoprim, Acute kidney injury, Serum creatinine

## Abstract

**Background:**

Sulfamethoxazole/trimethoprim (SMX/TMP) potentially increases the serum creatinine levels, resulting in acute kidney injury (AKI). However, the clinical characteristics of the AKI associated with SMX/TMP and the risk factors for its development have not been fully characterized.

**Methods:**

A retrospective cohort observational analysis was conducted on adult inpatients who started SMX/TMP treatment at the Tokyo Women’s Medical University, Yachiyo Medical Center, from April 2018 to March 2020. The primary outcome was AKI, defined as an increase in serum creatinine level of ≥ 50% from baseline. Multivariate logistic regression analysis was used to determine the risk factors for the AKI associated with SMX/TMP.

**Results:**

Of the 281 patients, 32 (11.4%) developed AKI. The multivariate logistic regression analysis identified that body mass index (BMI) (odds ratio [OR] = 0.86, 95% confidence interval [95% CI] 0.76–0.97, *p* < 0.01), presence of hypertension (OR = 2.69, 95% CI 1.11–6.49, *p* = 0.02), SMX/TMP daily dose (OR = 1.16, 95% CI 1.03–1.30, *p* = 0.02), and concomitant loop diuretic use (OR = 2.91, 95% CI 1.08–7.78, *p* = 0.04) were the associated risk factors for AKI in patients who were administered SMX/TMP.

**Conclusions:**

This study showed that low BMI, hypertension, high-dose SMX/TMP, and concomitant loop diuretic use increased the risk of AKI in patients administered SMX/TMP. Clinicians should consider monitoring the renal function in patients at a high risk of AKI.

## Background

Sulfamethoxazole/trimethoprim (SMX/TMP) is a combination of SMX and TMP in a ratio of 5:1. SMX/TMP blocks folate metabolism and exerts antifungal effects [1]. SMX/TMP is recommended for prophylactic and therapeutic use in the Japanese Domestic Guideline for Management of Deep-Seated Mycosis 2014 [2]. Generally, SMX/TMP is an acceptable selection for treating pneumocystis pneumonia (PCP) in high-risk patients such as immunocompromised hosts (e.g., those with connective tissue disease or a history of solid organ transplantation) [3–5].

SMX/TMP reduces the elimination of creatinine in urine via the inhibition of organic cation transporter 2, which elevates serum creatinine levels [6, 7]. The serum creatinine level following the administration of SMX/TMP is approximately 1.3 times higher than that at the onset of treatment; however, this increase is rarely a clinical problem and has been regarded as an apparent increase in serum creatinine level [6, 7]. However, there are some reports of acute kidney injury (AKI) caused by SMX/TMP [8–10]. As both SMX and TMP are mainly excreted via the kidney, dose adjustment of SMX/TMP is an important approach to avoid dose-dependent adverse events due to AKI. In addition, AKI is a known prognostic factor regardless of the underlying disease [11]. Previous reports have identified various risk factors for developing AKI associated with SMX/TMP, such as the presence of hypertension, diabetes, low baseline estimated glomerular filtration rate (eGFR), cardiac disorders, administration of high-dose SMX/TMP, and concomitant use of renin-angiotensin system blockers, potassium-sparing diuretics, and potassium supplements [8, 9, 12, 13]. However, it remains controversial about risk factors and no study has investigated the influence of loop diuretics on AKI associated with SMX/TMP.

This study aimed to identify the risk factors for AKI associated with SMX/TMP.

## Methods

### Study patients and design

A retrospective observational cohort analysis was conducted at the Tokyo Women’s Medical University, Yachiyo Medical Center. The eligibility criteria were hospitalized patients (≥ 20 years) who initiated SMX/TMP orally or intravenously between April 2018 and March 2020. Exclusion criteria were: 1) patients who had no data for serum creatinine level, or 2) patients who underwent renal hemodialysis, continuous hemodialysis, or peritoneal dialysis. The study protocol was approved by the Institutional Review Board of Tokyo Women’s Medical University (approval number: 5764) and was conducted in accordance with the 1964 Helsinki Declaration and its later amendments.

### Data collection

We extracted the following data from the electronic medical records: patient background (age, sex, height, body weight, and body mass index [BMI]), main diagnosis, comorbidities (diabetes and hypertension), clinical laboratory data (serum creatinine, eGFR, blood urea nitrogen [BUN], serum sodium, serum potassium, and serum chloride levels), details of SMX/TMP (purpose of administration, daily dose, and treatment duration), concomitant medications (non-steroidal anti-inflammatory drugs [NSAIDs], angiotensin-converting enzyme [ACE] inhibitors, angiotensin receptor blockers [ARBs], loop diuretics, β-lactams, glycopeptides, aminoglycosides, quinolones, anti-viral drugs, and calcineurin inhibitors). Hypertension (ICD 10 code: I10-15) and diabetes (ICD 10 code: E10-14), classified by the International Classification of Diseases 10, were determined from the information provided by the physician in the electronic medical records. We calculated the eGFR using the following prediction equation: eGFR = 194 × serum creatinine^−1.094^ × age^−0.287^ × 0.739 (if female) [14]. We evaluated the purpose of SMX/TMP administration based on the diagnoses registered by the physicians. For concomitant medications, we investigated representative medications reported to adversely affect the renal hemodynamics (NSAIDs, ACE inhibitors, ARBs, and loop diuretics) or cause renal toxicity (β-lactams, glycopeptides, aminoglycosides, quinolones, anti-viral drugs, and calcineurin inhibitors) [15, 16]. Clinical information was collected from the initial administration of SMX/TMP to the end of treatment or October 31, 2020, whichever was earlier.

### Outcome

The primary outcome was the occurrence of AKI, which was evaluated using the criteria from a previous report [8]. AKI was defined as an absolute increase in serum creatinine levels ≥ 50% during SMX/TMP treatment. If multiple AKI events occurred during the follow-up period, the first occurrence was considered the outcome. The Kidney Disease Improving Global Outcomes (KDIGO) diagnostic criteria [17] also defined AKI as an increase in serum creatinine by ≥ 0.3 mg/dL within 48 h. However, this definition was not used in this study because the data regarding the serum creatinine levels within 48 h after SMX/TMP administration were not available. Urine volume was not used for evaluation in this study because it was not measured in many cases, and it has been reported that the diagnosis of AKI by measuring urine volume may lead to overdiagnosis [18]. Because SMX/TMP has been associated with a decreased tubular secretion of creatinine without compromising glomerular filtration [6, 7], we simultaneously summarized the variations in BUN and BUN/serum creatinine in the AKI group to evaluate the cause of elevated serum creatinine levels.

### Statistical analysis

Statistical analyses were performed using JMP® Pro 15 (SAS Institute Inc., Cary, NC, USA). Statistical significance was set at *p* < 0.05, unless otherwise indicated.

Normally distributed continuous data were expressed as the mean ± standard deviation (SD) and compared using the Student’s t-test, whereas non-normally distributed data were expressed as the median and interquartile range (IQR) and compared using the Wilcoxon rank-sum test. Categorical data were expressed as numbers (%) and compared using the Pearson's χ^2^-test.

We performed a multivariate logistic regression analysis to identify the risk factors for AKI associated with SMX/TMP. The dependent variable was defined as AKI associated with SMX/TMP, and independent variables included patient backgrounds, such as age, sex, BMI, main diagnosis, comorbidity, clinical laboratory data, detailed SMX/TMP, and concomitant medications. In the univariable logistic regression analysis, independent variables with *p*-values < 0.10 were screened, and the stepwise forward selection method was used to determine the final model based on the Akaike information criterion at the minimum value. The main diagnosis, comorbidity, purpose of administration, and concomitant medications were used as categorical variables. When there was multicollinearity between any independent variables, we selected one of them with respect to the clinical aspect. The final model calculated the odds ratios (OR) and 95% confidence intervals (95% CI). Interactions between the independent variables were assessed using the final model.

## Results

### Study patients

**Fig. 1:**
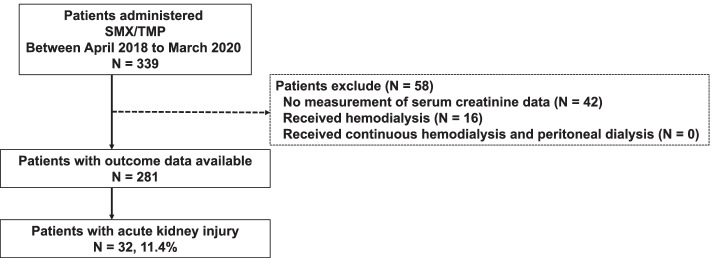
Flow chart of patient selection. SMX/TMP, sulfamethoxazole/trimethoprim; N, number

A flowchart of the patient selection process is shown in Fig. 1. There were 339 patients who started SMX/TMP at our institution between April 2018 and March 2020. We excluded 58 patients because the serum creatinine levels were not measured in 42 patients and 16 patients who were on hemodialysis. None of the patients had undergone continuous hemodialysis or peritoneal dialysis. The remaining 281 patients were included in the study.

The clinical data of the patients are summarized in Table 1. Of the study patients, 173 (61.6%) were men. The median patient age was 70 years (IQR range, 54–78 years). Most patients received 1600 mg /320 mg SMX/TMP per day for infections diseases, whereas all the patients who were administered SMX/TMP for prophylaxis received < 1600 mg /320 mg of SMX/TMP per day. Only 2 patients (0.71%) received SMX/TMP intravenously.

**Table 1:** Patient characteristics

	Non-AKI (*N* = 249)	AKI (*N* = 32)	*p*-value
**Patient background**			
Age, years	69 [53 − 78]	74 [64 − 80]	0.04^c^
Male gender, N (%)	151 (60.6)	22 (68.8)	0.37^a^
Height, cm	161.9 ± 10.4	158.4 ± 8.7	0.09^b^
Body weight, kg	58.4 [49.8 − 67.8]	50.2 [45.5 − 58.2]	< 0.01^c^
Body mass index, kg/m^2^	22.6 [19.7 − 25.4]	20.1 [18.3 − 22.0]	< 0.01^c^
**Main disease at admission**			
Infection, N (%)	43 (17.3)	10 (31.3)	0.06^a^
Cancer, N (%)	26 (10.4)	4 (12.5)	0.72^a^
Hematologic malignancy, N (%)	17 (6.8)	3 (9.4)	0.60^a^
Solid cancer, N (%)	9 (3.6)	1 (3.1)	0.89^a^
Rheumatic diseases, N (%)	21 (8.4)	1 (3.1)	0.29^a^
Interstitial lung disease, N (%)	44 (17.7)	3 (9.4)	0.24^a^
Nephrotic syndrome, N (%)	9 (3.6)	2 (6.3)	0.47^a^
Blood disease, N (%)	10 (4.0)	0 (0.0)	0.25^a^
Hepatitis, N (%)	14 (5.6)	0 (0.0)	0.17^a^
Renal transplantation, N (%)	49 (19.7)	2 (6.3)	0.06^a^
Others, N (%)	33 (13.3)	10 (31.3)	
**Comorbidity**			
Diabetes, N (%)	55 (22.1)	10 (31.3)	0.25^a^
Hypertension, N (%)	113 (45.4)	20 (62.5)	0.07^a^
**Clinical laboratory data**			
Serum creatinine, mg/dL	0.8 [0.6 − 1.1]	0.9 [0.6 − 1.2]	0.42^c^
eGFR, mL/min/1.73m^2^	71.7 ± 31.6	67.4 ± 28.6	0.46^b^
BUN, mg/dL	17.5 [13.3–23.7]	20.6 [13.3 − 29.4]	0.09^c^
Serum Na, mEq/L	137.6 ± 4.1	137.5 ± 4.8	0.87^b^
Serum K, mEq/L	4.1 [3.9 − 4.5]	4.2 [3.8 − 4.7]	0.74^c^
Serum Cl, mEq/L	104.8 ± 4.5	106.1 ± 5.7	0.19^b^
**Purpose of administration**			
Treatment, N (%)	73 (29.3)	19 (59.4)	< 0.01^a^
Pneumocystis pneumonia, N (%)	41 (16.5)	7 (21.9)	
Urinary tract infection, N (%)	15 (6.0)	6 (18.8)	
Bacterial pneumonia, N (%)	11 (4.4)	2 (6.3)	
Skin and soft tissue infection, N (%)	2 (0.8)	2 (6.3)	
Arthritis, N (%)	2 (0.8)	1 (3.1)	
Suppurative discitis, N (%)	1 (0.4)	0 (0.0)	
Psoas abscess, N (%)	0 (0.0)	1 (3.1)	
Pharyngitis, N (%)	1 (0.4)	0 (0.0)	
Prophylaxis, N (%)	176 (70.7)	13 (40.6)	
**SMX/TMP daily dose, mg**	400/80 [160/32–1600/320]	1600/320 [240/48–1600/32]	< 0.01^c^
Treatment duration, day	8 [5 − 15]	8 [5 − 16]	0.77^c^
**Concomitant medications**			
NSAIDs, N (%)	24 (9.6)	3 (9.4)	0.96^a^
ACEi/ARB, N (%)	46 (18.5)	6 (18.8)	0.97^a^
Loop diuretics, N (%)	29 (11.6)	11 (34.4)	< 0.01^a^
β-lactams, N (%)	75 (30.1)	16 (50.0)	0.02^a^
Glycopeptides, N (%)	3 (1.2)	3 (9.4)	< 0.01^a^
Aminoglycosides, N (%)	3 (1.2)	2 (6.3)	0.04^a^
Quinolones, N (%)	36 (14.5)	3 (9.4)	0.43^a^
Calcineurin inhibitors, N (%)	61 (24.5)	3 (9.4)	0.05^a^

Normally distributed data were expressed as mean ± SD, whereas non-normally distributed data were expressed as medians (interquartile ranges). Categorical data were expressed as numbers (%). AKI was defined as an increase in serum creatinine level of ≥ 50% from the baseline value

Other diseases included bronchial asthma, dysphagia, allergic skin disease, fracture, thyrotoxic storm, adrenocortical insufficiency, hydrocephalus, ulcerative colitis, gastrointestinal bleeding, and hyperglycemia and hypothermia

*AKI* Acute kidney injury, *N* Number, *eGFR* Estimated glomerular filtration rate, *BUN* Blood urea nitrogen, *Na* Sodium, *K* Potassium, *Cl* Chloride, *SMX/TMP* Sulfamethoxazole/trimethoprim, *NSAIDs* Nonsteroidal anti-inflammatory drugs, *ACEi* Angiotensin-converting enzyme inhibitor, *ARB* Angiotensin receptor blocker

^a^Pearson's χ^2^ test

^b^Student's t-test

^c^Wilcoxon rank-sum test

### Risk factors for AKI

Thirty-two (11.4%) patients who were administered SMX/TMP developed AKI within a median period of 8 days (IQR, 5–16 days) after the initiation of the treatment. The variations in serum creatinine, BUN, and BUN/serum creatinine ratio in the AKI group are summarized in Table 2. The BUN levels were measured in 30 patients in the AKI group. BUN was elevated by > 30% in 25 patients (83.3%) and decreased in only one patient. In the AKI group, 19 patients (63.3%) had a BUN/serum creatinine ratio of > 20 after SMX/TMP administration. None of the patients received concomitant antiviral drugs. Univariate logistic regression analysis revealed that age, BMI, hypertension, treatment for infection, SMX/TMP daily dose, concomitant loop diuretics, β-lactams, glycopeptides, and aminoglycosides were potential independent variables. Because of the multicollinearity between the purpose of administration and SMX/TMP daily dose (*r* = 0.83, *p* < 0.01, Spearman’s rank correlation coefficient was performed with prophylaxis = 0 and treatment = 1), we introduced the SMX/TMP dose to clarify the dose-dependent manner of AKI. There was no multicollinearity between the other independent variables. Using a stepwise forward selection method, we included the BMI, hypertension, SMX/TMP daily dose, concomitant loop diuretics, and glycopeptides as variables in the final model employed for assessing the risk factors for AKI after SMX/TMP administration. Multivariate logistic regression analysis demonstrated that BMI (per kg/m^2^) (OR = 0.86, 95% CI 0.76–0.97, *p* < 0.01), hypertension (yes) (OR = 2.69, 95% CI 1.11–6.49, *p* = 0.02), SMX/TMP daily dose (per SMX/TMP 400 mg/80 mg) (OR = 1.16, 95% CI 1.03–1.30, *p* = 0.02), and concomitant loop diuretic use (yes) (OR = 2.91, 95% CI 1.08–7.78, *p* = 0.04) were associated with AKI in patients administered SMX/TMP (Table 3). However, the influence of use of concomitant glycopeptides did not reach statistical significance (yes) (OR = 6.24, 95% CI 0.89–43.98, *p* = 0.07) (Table 3). The final model showed no interactions between the independent variables.

**Table 2:** Variation of serum creatinine, BUN, and BUN/serum creatinine in the AKI group

	Value	Increasing rate, %
**Serum creatinine, mg/dL (*N* = 32)**		
Baseline	0.9 [0.6 − 1.2]	65.9 [56.6 − 93.4]
Onset of AKI	1.5 [1.1 − 2.1]	
Increasing value	0.6 [0.4 − 1.0]	
**BUN, mg/dL (*N* = 30)**		
Baseline	20.7 [13.8 − 29.3]	61.6 [39.2 − 115.0]
Onset of AKI	38.2 [22.5 − 53.9]	
Increasing value	12.3 [6.9 − 27.5]	
**BUN/serum creatinine (*N* = 30)**		
Baseline	23.7 [19.1 − 28.9]	− 5.6 [− 24.2 − 9.6]
Onset of AKI	22.8 [17.4 − 29.4]	
Increasing value	− 1.3 [− 7.1 − 1.9]	

Data are expressed as median (interquartile range)

*BUN* Blood urea nitrogen, *AKI* Acute kidney injury, *N* Number

**Table 3:** Multivariate logistic regression analysis of risk factors for AKI

Independent variables	Univariate			Multivariate		
	OR	95% CI	*p* value	OR	95% CI	*p* value
Age, per year	1.03	1.00 − 1.06	0.02			
Body mass index, per kg/m^2^	0.86	0.77 − 0.96	< 0.01	0.86	0.76 − 0.97	< 0.01
Hypertension (yes)	2.01	0.94 − 4.28	0.07	2.69	1.11 − 6.49	0.02
Treatment for infection (yes)	3.52	1.65 − 7.51	< 0.01			
Daily dose, per SMX400 mg/TMP80 mg	1.13	1.02 − 1.25	0.02	1.16	1.03 − 1.30	0.02
Loop diuretics (yes)	3.97	1.74 − 9.08	< 0.01	2.91	1.08 − 7.78	0.04
β-lactams (yes)	2.32	1.10 − 4.88	0.03			
Glycopeptides (yes)	8.48	1.64 − 43.99	0.02	6.24	0.89 − 43.98	0.07
Aminoglycosides (yes)	5.47	0.88 − 34.04	0.09			

We summarized the independent variables with < 0.10 in the univariate logistic regression analysis

Of 281 patients, 32 had AKI and 249 did not have AKI

*AKI* Acute kidney injury, *OR* Odds ratio, *95% CI* 95% confidence interval, *SMX* Sulfamethoxazole, *TMP* Trimethoprim

## Discussion

Our study identified that the risk factors for AKI in patients administered SMX/TMP were low BMI, hypertension, high-dose SMX/TMP, and concomitant use of loop diuretics. Clinicians should consider monitoring the renal function and managing several factors such as fluid balance and the use of concomitant medications during SMX/TMP treatment.

The incidence of AKI associated with SMX/TMP differs according to the patient background. For instance, a previous report demonstrated that the incidence of nephrotoxicity for SMX/TMP was extremely low (< 0.01%) in an outpatient setting [10]. In contrast, Fraser et al. [8] and Rajput et al. [9] reported that the incidences of AKI associated with SMX/TMP in hospitalized patients were 11.2% and 19.6%, respectively. In particular, Fraser et al. [8] reported that most patients were administered low doses (SMX/TMP 800 mg/160 mg), and there were significantly more hypertensive patients in the AKI group, which supports our findings. Therefore, clinicians should understand the patient’s background. The extent of elevated BUN in renal injury due to SMX/TMP remains unclear. However, our study showed that BUN increased concomitantly with creatinine in nearly all cases. This suggests that renal failure, rather than inhibition of tubular secretion of creatinine without a compromised glomerular filtration, is a more common cause of increased serum creatinine levels in patients taking SMX/TMP.

Previous reports indicated hypertension, diabetes, low baseline eGFR, cardiac disorders, high-dose SMX/TMP, concomitant use of renin-angiotensin system blockers, potassium-sparing diuretics, and potassium supplements are risk factors for AKI associated with SMX/TMP [8, 9, 12, 13]. However, our study examined whether loop diuretics could increase the risk of AKI. In this study, as in previous reports, hypertension and high-dose SMX/TMP were identified as risk factors [8, 12], although low BMI and concomitant administration of loop diuretics were also identified as independent risk factors.

The relationship between body size and AKI associated with SMX/TMP has not been well investigated. Obesity has a significant impact on the development of AKI [19]. In contrast, our study showed the opposite. Of the 37 patients with BMI < 18.5 kg/m^2^ in the present study, 9 (24.3%) were treated for PCP, which was more common in these patients than in patients with BMI > 30 kg/m^2^ (5.4%) (data not shown). Weight loss is one of the major symptoms in patients infected with *Pneumocystis jirovecii*, indicating that the PCP patients in our study tended to be underweight [20]. PCP was treated with high-dose SMX/TMP, and we consider that the low BMI resulted in relatively increased blood levels, thus adversely influencing the occurrence of AKI.

Hypertension with systolic blood pressure ≥ 140 mmHg is a risk factor for AKI associated with SMX/TMP [8]. In contrast, James et al. [21] showed that the development of AKI was significantly associated with the presence of urinary protein and low renal function associated with hypertension but not with the severity of hypertension. Although the data on blood pressure could not be collected, we consider that hypertension may be a risk factor for AKI associated with SMX/TMP, regardless of the severity of hypertension. In addition, representative antihypertensive medications, such as ACE inhibitors and ARBs, are known to reduce intraglomerular pressure, resulting in AKI [15]. However, no significant differences were observed in AKI development. This is because 170 (60.4%) patients did not meet the diagnosis of chronic kidney disease, indicating that the patients could tolerate the renal ischemia caused by ACE inhibitors and ARBs.

Some reports have indicated that the incidence of AKI was higher in patients who were administered high-dose SMX/TMP than in those administered a normal dose [12, 22]. However, these previous reports [12, 22] showed that the incidence of AKI was 2% to 4% in patients receiving a high dose, which was lower than that in our results. These previous reports [12, 22] recruited outpatients and administered treatments for conditions such as skin or soft tissue infections. In contrast, our study was conducted on inpatients and may have been more severe than those in previous reports [12, 22]. Our study supports the finding that high-dose SMX/TMP is a risk factor for AKI, regardless of the severity.

Loop diuretics cause renal ischemia by decreasing the fluid volume, which causes pre-renal AKI. In addition, the accumulation of SMX/TMP in the blood due to loop diuretics may cause secondary AKI. In our study, the proportion of patients with a BUN/serum creatinine ratio > 20 was 66.7% in the AKI group. Since a BUN/serum creatinine ratio > 10 generally indicates a tendency for dehydration, there is a possibility of SMX/TMP accumulation due to dehydration. Although several reports have demonstrated that SMX/TMP causes interstitial nephritis and post-renal AKI owing to the crystallization of SMX [23–25], we consider that pre-renal AKI should also be carefully monitored. In addition, potassium-sparing diuretics have been reported to cause AKI associated with SMX/TMP [13], indicating that dehydration causes AKI, regardless of the type of diuretic used. Attention should be paid to dehydration caused by the combination therapy of SMX/TMP with loop diuretics.

This study had several limitations. First, this was a single-center retrospective observational study, and a potential selection bias could not be avoided. Further analysis should be conducted to clarify the effect of comorbidities, such as cancer or cardiac disorders, on AKI associated with SMX/TMP. Second, KDIGO defined AKI as 1) an increase in serum creatinine level by ≥ 0.3 mg/dL within 48 h of initiation of therapy, 2) an increase in serum creatinine to ≥ 1.5 times baseline within the previous 7 days, and 3) urine volume ≤ 0.5 ml/kg/hour for 6 h [17]. However, we could not collect sufficient data on serum creatinine levels within 48 h of initiating SMX/TMP therapy and the urine volume, so our study may not have adequately diagnosed AKI. Third, we could not assess the severity of sepsis based on the sequential organ failure assessment score because we could not collect data on the level of consciousness. Severe sepsis has been reported to affect renal function [26], and its severity may have influenced the results of this study as well. Fourth, we found that concomitant loop diuretics were a risk factor for AKI in patients administered SMX/TMP. However, we could not rule out the possibility of AKI due to loop diuretics. Fifth, some of the cases were missing, and the sample size of patients with concomitant use of glycopeptides and aminoglycosides was small; hence, we could not assess the relationship between AKI associated with SMX/TMP and these medications. Finally, although we also attempted to analyze the impact of the human immunodeficiency virus on AKI; there were no patients with the human immunodeficiency virus infection.

## Conclusions

The risk factors for AKI in patients who were administered SMX/TMP are multifactorial, including low BMI, hypertension, high-dose SMX/TMP, and concomitant loop diuretic use, indicating that clinicians should manage these factors to avoid AKI. It is important to consider fluid management and to modify the use of concomitant medications in settings requiring administration of high-dose SMX/TMP.

## Data Availability

The datasets used and/or analyzed during the present study are available from the corresponding author upon reasonable request.

## Abbreviations

SMX/TMP: Sulfamethoxazole/trimethoprim
PCP: Pneumocystis pneumonia
AKI: Acute kidney injury
eGFR: Estimated glomerular filtration rate
BMI: Body mass index
BUN: Blood urea nitrogen
NSAIDs: Non-steroidal anti-inflammatory drugs
ACE: Angiotensin-converting enzyme
ARBs: Angiotensin receptor blockers
KDIGO: Kidney Disease Improving Global Outcomes
